# Allostery without Conformational Change: A Native Mass Spectrometry Perspective

**DOI:** 10.1021/acs.jpcb.5c03261

**Published:** 2025-08-19

**Authors:** He Mirabel Sun, Kacie A. Evans, Morgan Powers, Zhenyu Xi, Carter Lantz, Arthur Laganowsky, Hays Rye, David H. Russell

**Affiliations:** † Department of Chemistry, 14736 Texas A&M University, College Station, Texas 77843, United States; ‡ Department of Biochemistry and Biophysics, Texas A&M University, College Station, Texas 77843, United States

## Abstract

Native electrospray ionization-mass spectrometry (nESI-MS) enables studies of intact proteins, protein complexes, and protein–ligand complexes. Variable temperature (vT)-nESI-MS, where the temperature of the solution contained in the ESI emitter can be varied from 2 to 100 °C, adds new capabilities for dissecting the thermodynamics for protein–ligand binding. Here, vT-nESI-MS and ion mobility spectrometry (IMS) are used to compare the effects of temperature and nESI buffers on nucleotide (ADP) binding for the GroEL single ring mutant (SR1). Temperature-dependent shifts for average charge states (*Z*_avg_) and rotationally averaged collision cross sections (CCS) for both apo- and nucleotide-bound SR1 complexes (SR1-ADP_*n*_, *n* = 1–7) indicate that nESI buffers alter structure, stabilities, and dynamics. These studies report nucleotide (ADP) binding affinities (*K*_a_) and insight into cooperativity and enthalpy–entropy compensation (EEC). Specifically, we focus on three commonly used native ESI buffers: ammonium acetate (AmAc), triethylammonium acetate (TEAA), and ethylenediammonium acetate (EDDA). In AmAc solutions, ADP binding is highly cooperative at low temperatures (2–21 °C) but is significantly diminished at higher temperatures (21–31 °C). While cooperative ADP binding is only observed at low temperatures (4 °C) for TEAA solutions, it is absent in EDDA solutions. Collectively, these findings illustrate very different influences of ammonium and alkyl ammonium ions on the SR1 conformation and dynamics as manifested by changes in *Z*_avg_ (change of solvent-accessible surface area) and thermodynamics for nucleotide binding. Moreover, temperature-dependent changes in *Z*_avg_ and ligand binding provide additional experimental data that support prior work on the effects of hydration on cold protein folding. These results also align with recent computational work for the effects of hydration water on protein binding sites as well as membrane protein complex-lipid binding. The observed temperature-dependent changes in *Z*_avg_, buffer-dependent nucleotide binding, EEC, and changes in heat capacity strongly suggest that ADP influences the conformational states of the SR1 complex. Note, however, that large-scale structural changes in the SR1 complex are not observed in the IMS CCS experiments. Collectively, these results suggest that ADP binding alters key structural and/or dynamic properties of SR1, changes that are not observed in the overall, macroscopic structure of the complex. We suggest that SR1-ADP binding is an archetypal example of “allostery without (measurable) conformational change”.

## Graphical abstract

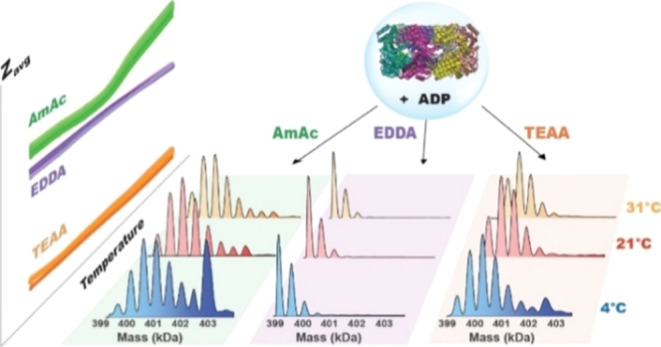

## Introduction

Native electrospray ionization-mass spectrometry (nESI-MS) has evolved from analysis and characterization of secondary, tertiary, and quaternary structures of intact proteins and protein complexes to studies of physicochemical properties, stabilities, and dynamics, including their interactions with metals, small molecules, and other biomolecules.^1–4^ Recent advances in nESI-MS are made possible by increased sensitivity, mass resolution, and mass range of MS instruments specifically designed for studies of large biomolecules.^2,5^ These improvements in instrumentation further facilitate kinetics and thermodynamics studies, viz., slow mixing mode (SLOMO) nanoflow nESI-MS,^3,6^ hydrogen–deuterium exchange (HDX-MS),^7^ and protein cross-linking.^8^ Moreover, variable temperature (vT)-nESI has allowed studies on the effect of temperature (cool and heat, 2–95 °C) of the solution in the ESI emitter.^9–11^ As interest in and applications of nMS grow, increasing efforts are made to demonstrate that nESI-MS experiments are capable of capturing solution-phase states of proteins and protein complexes and how changes in structure(s), stabilities, and dynamics are influenced by the presence of other species, e.g., ligands,^10,12–15^ cofactors,^16,17^ and other proteins/nucleic acids,^18^ including the effects of hydration.^19–22^ Oftentimes, these reactions promote changes in the solvent accessible surface area (SASA), a conformational change that affects the average charge state and/or collision cross section (CCS) that can be detected by ion mobility spectrometry (IMS).^23–30^ For complex systems that involve binding of multiple ligands, vT-nESI-MS analysis can resolve binding of one ligand at a time,^31–34^ thereby dissecting specific reaction channels, including changes in enthalpy–entropy compensation for individual binding sites.^35^ Recently, a study showed that the role of hydration in protein complex stability can also be interrogated by vT-nESI-MS that complements high-pressure NMR experiments.^36,37^ These unique features of vT-nESI-MS add new capabilities for studies in areas of drug discovery and structural biology.^38–41^

Recent studies have shown that structure, stabilities, dynamics, and ligand binding are directly linked to the presence of volatile electrolytes that mimic solution properties experienced by proteins under physiological conditions, commonly referred to as “ESI buffers”. The most widely used nESI buffer is ammonium acetate (AmAc). Konermann has noted that AmAc has limited pH buffer capacity;^42^ however, AmAc plays a crucial role in nESI-MS by providing adequate ionic strength and preventing undesired adducts.^43^ The advantages for using AmAc solutions were first described by Kebarle and co-workers,^44^ and this mechanism implies that at least some fraction of the ammonium and/or acetate ions are in direct contact with surface-exposed hydrated hydrophilic groups, which are subject to temperature changes and alter hydration of the protein. Rapid evaporation of buffer and water during transition of the ions from solution to the gas phase captures solution-phase structures of the protein and protein–ligand complexes.^45,46^ ESI solutions that contain alkyl ammonium ions (AkAm) yield relatively narrow distributions of lower charge state ions by a mechanism referred to as charge reduction reactions.^47–49^ Alkylamines are more basic than ammonia owing to the inductive effect of the alkyl groups,^50^ and this increase in basicity favors proton abstraction from the protein by the departing alkylamine. The ESI mass spectra with AkAm buffers are similar to those obtained using AmAc; however, the lower charge states provide evidence that charge reduction reactions can have direct effects on stabilities, dynamics, and conformation of the protein ions.^47,51^ Zenobi and co-workers noted that some complexes are more stable when generated from AkAm solutions compared to AmAc solutions, and in some cases, dissociation constants (*K*_d_) for protein–ligand complexes were ∼40% lower in AkAm solutions compared to AmAc solutions.^47^ Walker et al. showed that both solution temperature and native MS buffers have significant effects on GroEL (tetradecamer), and the different charge states exhibit different reactivities and thermodynamics for nucleotide (ATP) binding.^10,33^ Most notable were the very different effects that solution temperature and AmAc versus ethylenediamine diacetate (EDDA) had on the enthalpy–entropy compensation (EEC) for GroEL tetradecamer ATP binding. Kumar et al. also showed that protein–lipid interactions are influenced by detergents and charge reducing reagents,^52^ and Wysocki reported that lower ion charge states are “more native.”^51^

We previously reported on the influence of temperature on the stabilities, ligand binding, and stoichiometry of GroEL, GroEL-ATP, and GroEL-GroES complexes.^10^ A more recent study reported detailed thermodynamics for nucleotide (ATP and ADP) binding to the GroEL tetradecamer.^33^ The conformational changes of GroEL in response to nucleotide binding have also been well characterized structurally.^53–57^ Liebermann et al. reported that GroEL can populate several microstates in solution that shift between states in response to solution conditions.^58^ Using SR1 as a model, Liebermann et al. employed single-molecule Förster resonance energy transfer (smFRET) to identify four conformational microstates of the GroEL SR1 mutant (E255C/D428C), which interconvert on the millisecond time scale.^58^ Importantly, the population of conformationally expanded microstates is altered upon binding ATP, suggesting that upward motion of the apical domains favors an open conformation. In addition to conformational microstates, proteins such as GroEL also access a range of protonation microstates, where changes in charge state distribution can significantly alter stability and allostery, as demonstrated by Monte Carlo sampling.^59^ We anticipate that the application of vT-nESI-MS-enabled charge state and thermodynamics analysis to this system will provide even more detailed insight into these nucleotide-dependent transitions and, in particular, how solution conditions impact the free-energy landscape (FEL) of GroEL.

In prior work on GroEL, we found that the presence of ammonium ions in AmAc and EDDA buffers enhanced GroEL ATPase activity.^33^ While this behavior is consistent with known effects of monovalent ions (K^+^, Rb^+^, and NH_4_^+^) on the hydrolysis of ATP by GroEL, there are no prior studies on the effects of AmAc, triethylammonium acetate (TEAA), or EDDA as ESI buffers. Moreover, the striking differences in GroEL-nucleotide binding profiles and EEC in AmAc versus EDDA bring forth the need for further investigations of the effects of native MS buffers on proteins and protein complexes. EEC reports changes in enthalpy and entropy that are linearly correlated in many chemical and biological processes, especially for ligand binding reactions and molecular recognition. While the interpretation of EEC data for large biomolecule-ligand binding is complex, it is especially challenging for GroEL tetradecamer-nucleotide binding owing to the negative inter-ring cooperativity between the two heptameric rings of the nested-cooperativity model.^60,61^ In order to avoid these complications, this study focused on the SR1 GroEL variant, a heptamer, and the binding of ADP to avoid ATP hydrolysis.^33^ Here, we use vT-ESI-nMS to collect ADP binding information on SR1 GroEL under different buffer conditions. The resulting average charge state (*Z*_avg_), rotationally averaged collision cross sections (CCS), and thermodynamic information obtained from the binding experiments provide evidence that buffer molecules interact with SR1 GroEL, altering the distribution of protonation microstates in solution and the extent of allosteric regulation.

## Methods

### Sample Preparation

All chemicals, including magnesium acetate (MgAc_2_), ADP, AmAc, EDDA, and TEAA were purchased from Sigma-Aldrich (St. Louis, MO) and dissolved in LC–MS grade deionized water. SR1 was overexpressed in *E. coli* as described previously.^62^ ADP sample aliquots with 1 mM MgAc_2_ were stored at −20 °C and freshly diluted with 200 mM AmAc (pH 6.8), EDDA (pH 6.3), or TEAA (pH 7) buffer containing 1 mM MgAc_2_, then added to protein prior to analysis. Protein concentration was measured by using UV–vis at 280 nm. Fresh SR1 was diluted 3-fold and buffer exchanged into corresponding buffer containing 1 mM MgAc_2_ by using a Micro Bio spin *P*6̅ gel column (Bio-Rad).

### Variable-Temperature Native Mass Spectrometry Analysis

The temperature of the solution contained in the nano-ESI emitter was controlled by the home-built variable temperature device as described previously.^9^ The vT-ESI temperature suggests an error of ±1.5 °C. Solution temperatures used for this study were 4–45 °C, but the data for *Z*_avg_ and thermodynamics was limited to 5–35 °C. ADP solutions at various concentrations prepared in the same buffer as that for SR1 were titrated into SR1 and incubated at each temperature for 1 min. Then, raw mass spectra were collected on a Thermo Q Exactive UHMR (ultrahigh mass range) hybrid quadrupole orbitrap mass spectrometer. The resolution setting was maintained at 25,000 with 5 microscans for SR1-ADP binding experiments in AmAc and 12,500 for SR1-ADP binding in EDDA and TEAA. The capillary temperature was set to 120 °C with in-source trapping set to −200 V, and the HCD energy was set to 150 V. Using these conditions, no gas-phase dissociation products were observed. The acquisition time for each spectrum was set to 1 min.

### CCS Measurement

The CCS of SR1 and SR1-ADP binding products were measured on the custom periodic focusing Fourier transformation drift tube ion mobility spectrometer coupled to the Thermo Q-Exactive UHMR MS as described in detail previously.^63,64,74^ Ion mobility measurement was conducted on samples containing 1 μM SR1 and 1 mM MgAc_2_ in 200 mM AmAc, EDDA, or TEAA. The reported CCS values and IM profiles are the average of triplicate measurements. The drift gas was helium at 1.48 Torr.

### Data Processing

Unidec was used to assign the charge states, mass, and abundance of each individual species detected in the mass spectra. *Z*_avg_ was calculated as the weighted average of all charge states for a mass species. The integrated signal intensities of each complex were used to fit a sequential binding model for solving dissociation constant (*K*_d_) values as previously described by Cong et al.,^31^ from which the apparent binding constants (or the equilibrium constant) *K*_eq_ are obtained as the reciprocals. The intrinsic binding constants (*K*_a_) are obtained using eq 1, where *N* is the total number of binding sites and *i* is the number of bound nucleotides.

1 $$K_{a}=K_{eq}\times \frac{i}{N-i+1}$$

The Gibbs free energy for ADP binding can be calculated by using eq 2. Enthalpy and the change in heat capacity for the binding reaction at the temperature *T*_0_ was derived with the nonlinear van’t Hoff equation^66^ (eq 3), which includes a constant temperature-dependent heat capacity change.

2 $$\Delta G=-RT\ln K_{a}$$

3 $$\mathrm{Ln}K_{a}=\mathrm{Ln}K_{0}+\frac{\Delta C_{p}}{R}\cdot \mathrm{Ln}\frac{T}{T_{0}}+\left(\frac{\Delta H_{0}-T_{0}\cdot \Delta C_{p}}{R}\right)\cdot \left(\frac{1}{T_{0}}-\frac{1}{T}\right)$$

*R = 8.314J·K^–1^·mol^–1^, *K*_0_ is the intrinsic binding constant at *T*_0_.

The magnitude of Δ*S* at *T*_0_ can then be calculated from the Δ*H*_0_ and Δ*G*_0_ values using the following equation:

4 ΔG=ΔH−TΔS

## Results

In this work, we compare the influence of the temperature of the solution contained in the ESI emitter for three commonly used native ESI buffers (AmAc, EDDA, and TEAA) on *Z*_avg_, ion mobility, CCS, and thermodynamics for SR1-ADP binding. It is anticipated that these changes in the solution conditions will yield significantly different distributions for SR1 and SR1-ADP protonation microstates.^58^ Prior vT-ESI-MS studies on the GroEL tetradecamer illustrated that ESI buffers and solution temperature induce marked changes of *Z*_avg_, CCS, and thermodynamics (*K*_a_, EEC, and van’t Hoff analysis).^33^ Here, we use vT-ESI-MS-IMS to better understand the role of solution parameters, e.g., temperature, pH, and native MS buffers, on solution-phase chemical reactions, viz., protein–ligand binding affinities (*K*_a_) and EEC.

Figure 1 contains plots showing temperature (5–35 °C)-dependent *Z*_avg_ changes for SR1 and SR1-ADP_*n*_ (*n* = 1–7) in AmAc, TEAA, and EDDA solutions contained in the ESI emitter. At low temperatures (5–20 °C), *Z*_avg_ for apo SR1 and SR1-ADP_*n*_ (*n* = 1–7) undergo small decreases, but at temperatures greater than ∼20 °C, *Z*_avg_ changes in AmAc solutions are markedly different from those observed for TEAA and EDDA solutions. Thermal decomposition of SR1 and SR1-ADP complexes is observed in all three buffers at temperatures greater than 40 °C (Figure S2); similar behavior was also observed for the GroEL tetradecamer at *T* > ∼52 °C.^10^ Owing to the low abundances of SR1-ADP complexes, *Z*_avg_ data for TEAA and EDDA solutions were also acquired for solutions containing higher concentrations of ADP (Figure 1C,E). For both TEAA and EDDA solutions at low temperatures (5–20 °C), changes in *Z*_avg_ are similar to SR1 and SR1-ADP_*n*_ in AmAc solutions; however, at temperatures greater than 20 °C, *Z*_avg_ decreases in EDDA (Figure 1D,E) but not TEAA (Figure 1B,C). *Z*_avg_ reduction upon increasing the temperature is an atypical behavior. Note that the *Z*_avg_ for SR1-ADP_6, 7_ in AmAc (Figure 1A) and TEAA (Figure 1C) are lowered in comparison to SR1-ADP_0–5_, a feature also observed for SR1-ADP_7_ in EDDA (Figure 1E) at 20–35 °C. Figure 1F contains ion mobility CCS data for each of the charge states of SR1 and SR1-ADP_7_ in AmAc, EDDA, and TEAA (IM profiles shown in Figure S3). While the CCS for both SR1 and SR1-ADP are similar, the CCS obtained from EDDA solution are significantly larger, as would be expected for a more extended conformation (vide infra).

**Figure 1:**
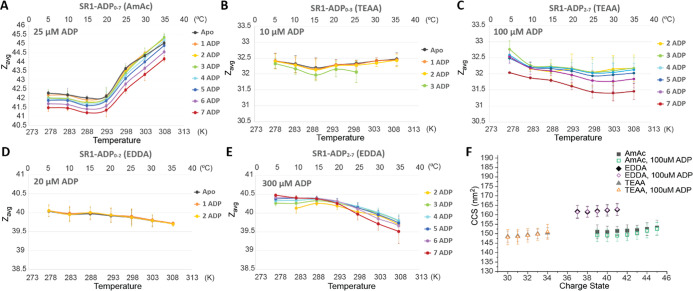
(A–E) Effects of solution temperature contained in the ESI emitter on the *Z*_avg_ for SR1 and SR1-ADP_*n*_ complexes in 200 mM AmAc, EDDA, and TEAA buffers containing 1 mM MgAc_2_, 1 μM SR1, and various concentrations of ADP. 20 μM ADP was present in the AmAc solution (A). Owing to low abundances for SR1-ADP complexes at low ADP concentrations, the *Z*_avg_ changes for SR1 in TEAA were obtained at (B) 10 μM and (C) 100 μM ADP concentrations; similarly, data for EDDA were obtained at 20 μM (D) and 300 μM (E) ADP concentrations. The averaged data were generated from triplicate measurements. Collision cross sections for SR1 and SR1-ADP_*n*_ are shown in (F) with the error bar showing the peak widths.

Deconvoluted mass spectra for SR1-ADP binding in AmAc, EDDA, and TEAA acquired at temperatures of 5, 21, and 31 °C are shown in Figure 2(A,C). Mass assignment data are contained in the Supporting Information (Tables S1 and S2). The masses of the SR1-ADP-bound species reported in Table S3 involve some discrepancies from the expected mass addition of ADP and Mg^2+^ (451 Da) due to retained salts and water molecules during the ESI process.^67^ In AmAc and TEAA solutions, the abundance of SR1-ADP_7_ is increased at low temperatures and decreased at high temperatures, similar to that observed for GroEL tetradecamer binding ATP,^33^ but ADP binding in EDDA is much less affected by temperature. The influence of temperature on ADP binding is reversible, as illustrated in Figure 2(A–C). Our observation of cooperativity in ADP binding to SR1 is consistent with prior findings by Poso et al.^68^ and contrasts with earlier reports suggesting noncooperative behavior, likely due to methodological differences and ensemble averaging effects inherent to fluorescence-based arrays.^69^ Data acquired for EDDA solutions at higher concentrations of ADP (300 μM) yield higher abundances of SR1-ADP_3–5_, while the low-to-high and high-to-low temperature cycles yield very similar abundances of SR1-ADP_*n*_ complexes (see Figure S1). Figure 2D–F shows the mole fractions of individual SR1-ADP_*n*_ (*n* = 0–9) complexes at temperatures of 5, 21, and 31 °C in the three buffers. It is interesting to note that in AmAc and TEAA, nonspecific binding products SR1-ADP_8,9_ are observed at ADP concentrations higher than 30 μM, with higher abundance in AmAc and at 4 °C. Figure 2(G–I) shows binding constants (*K*_a_) for each SR1-ADP binding reaction calculated using the relative intensities of individual mass species as previously reported by Cong et al.^31^ The binding constants are statistically corrected to account for the number of modes in which ligands may associate or dissociate from the complex using eq 1 described in the Methods section as reported in our previous studies.^33,65,70^ Binding constants corrected for nonspecific binding were calculated as described by Horovitz^71^ and are summarized in Table S3. After correction, AmAc and TEAA showed only minor deviations. In contrast, EDDA data indicated that ADP binding events 5–7 were likely nonspecific. Applying the nonspecific binding model yielded poor fits beyond the fourth binding, with affinities falling below the nonspecific constant, resulting in high fitting error. Nonetheless, for this study, we proceeded under the assumption that all seven potential ADP binding sites remain available. Note that AmAc and TEAA solutions are the only systems where the binding of 7 ADPs is abundant. Moreover, AmAc solutions exhibit greater binding cooperativity, and this is observed only at low (4 °C) temperatures; this behavior is very similar to that observed for GroEL tetradecamer, e.g., cooperative binding for the 14th ATP at 5 °C.

**Figure 2:**
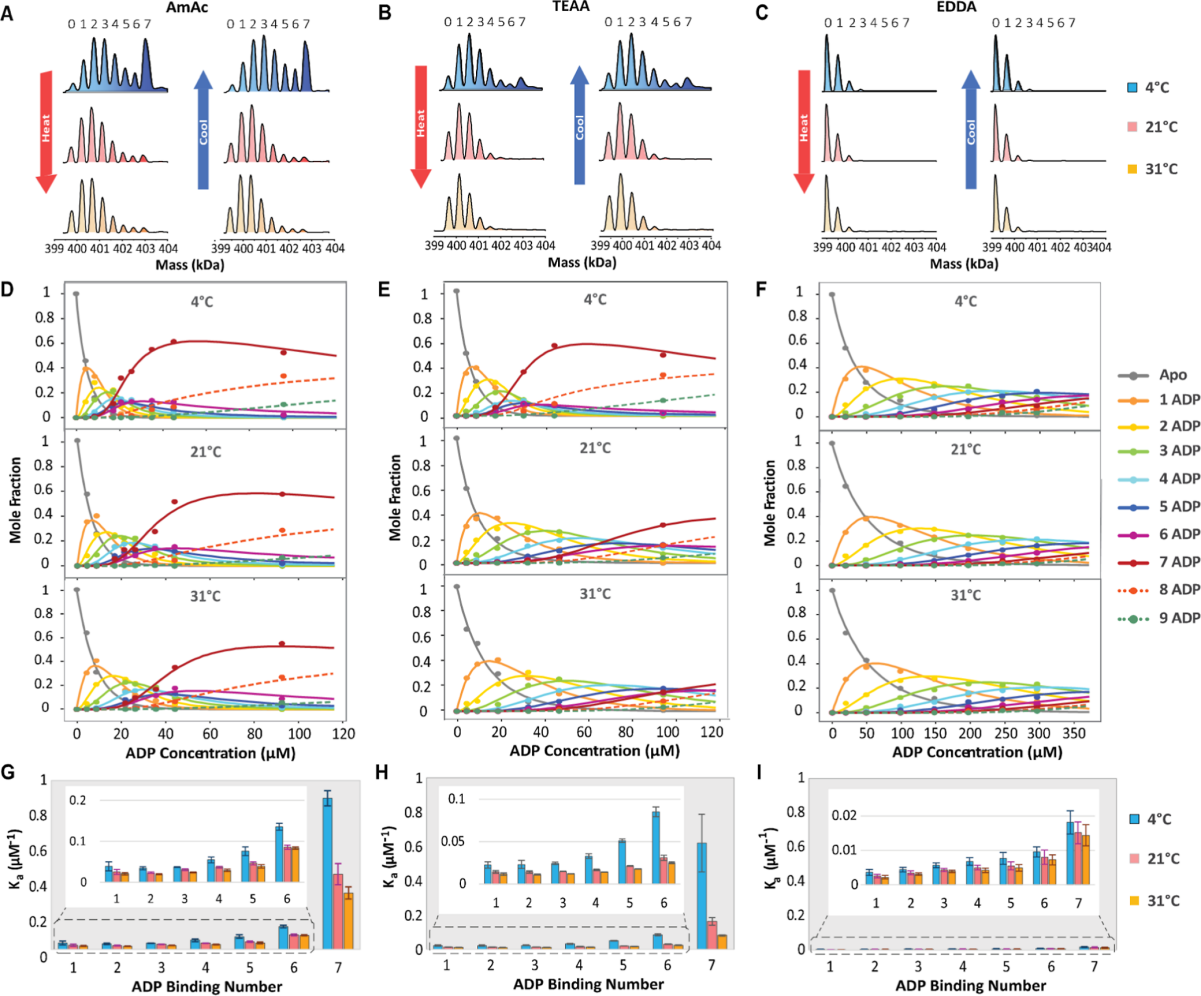
(A–C) Deconvoluted mass spectra of ADP binding products of 1 μM SR1 in 200 mM AmAc, TEAA, and EDDA buffer containing 1 mM MgAc_2_ and 20 μM ADP at cold (4 °C), medium (21 °C), and high (31 °C) temperatures. Note that each set of spectra compares the result of heating (4–31 °C) and cooling (31–4 °C) the solution contained in the ESI emitter. (D–F) Mole fraction plots for SR1-ADP_*n*_ (*n* = 0–9) complexes in AmAc, TEAA, and EDDA buffer at low (4 °C), medium (21 °C), and high (31 °C) temperatures at varying ADP concentrations. Note that the *x*-axis has a larger range for EDDA. Nonspecific ADP binding products SR1-ADP_8–9_ are observed at high ADP concentrations. (G–I) The bar charts show intrinsic binding constants (*K*_a_) for individual SR1-ADP binding steps at 4 °C (blue), 21 °C (red), and 31 °C (yellow) in 200 mM solutions of AmAc, TEAA, and EDDA, respectively. All binding constants are generated from triplicated data sets, and error bars are standard deviations of the three replicates.

Van’t Hoff analysis of the data shown in Figure 2 was used to evaluate the thermodynamics (Δ*G*, Δ*H*, and -*T*Δ*S*) at 25 °C for each of the ADP binding reactions (Figure 3). In each buffer, the changes in free energy are rather small, but the changes in enthalpy and entropy vary, demonstrating EEC, a phenomenon also observed in GroEL-ATP binding.^33^ The EEC patterns are similar for AmAc and EDDA solutions, except for the seventh ADP binding. In both cases, ADP binding is entropically favored, with the exception of binding of the seventh ADP in AmAc; very similar behavior was observed for the GroEL-ATP system. EEC for TEAA solutions shows exactly opposite behavior in that ADP binding is favored by decreasing entropy until the binding of the seventh ADP, which has a significant unfavorable entropy and large favorable enthalpy. In AmAc, where ADP exhibits the highest binding affinity and cooperativity, the thermodynamics for individual ADP binding exhibit favorable EEC for the first 5 ADP binding reactions. Note, however, that the EEC for binding of the sixth and seventh ADP are very different for AmAc and TEAA, featuring more favored enthalpy (Figure 3D and 3E). For EDDA, the specific binding model produced consistent thermodynamic patterns for the fifth–seventh ADP binding events, characterized by entropy-dominated binding with minimal enthalpic contributions, features commonly associated with nonspecific interactions. In our data, enthalpy contributions progressively decreased across these binding steps in EDDA. Therefore, we report all seven binding events using the specific binding model, with the nonspecific fit shown in Figure S5. It is interesting, however, to note the very different EEC behaviors for EDDA (Figure 3F). The simplest explanation of the observed trends for EEC is that these events are reporting on the full commitment of the SR1 ring to the allosteric transition, a shift that is inhibited in EDDA buffer. As seen in the compensation plots (Figure 3G–I) AmAc and TEAA display similar slopes, implying comparable conformational and hydration changes in SR1, whereas the slope for EDDA is different, consistent with altered or restricted structural transitions or solvent rearrangements. This interpretation is supported by prior work demonstrating that solvation changes play a central role in EEC, especially when protein conformational shifts are coupled to rearrangements in the hydration sphere.^72^ Collectively, these results suggest that EDDA imposes constraints on the SR1 conformational restructuring as ADP binds.

**Figure 3:**
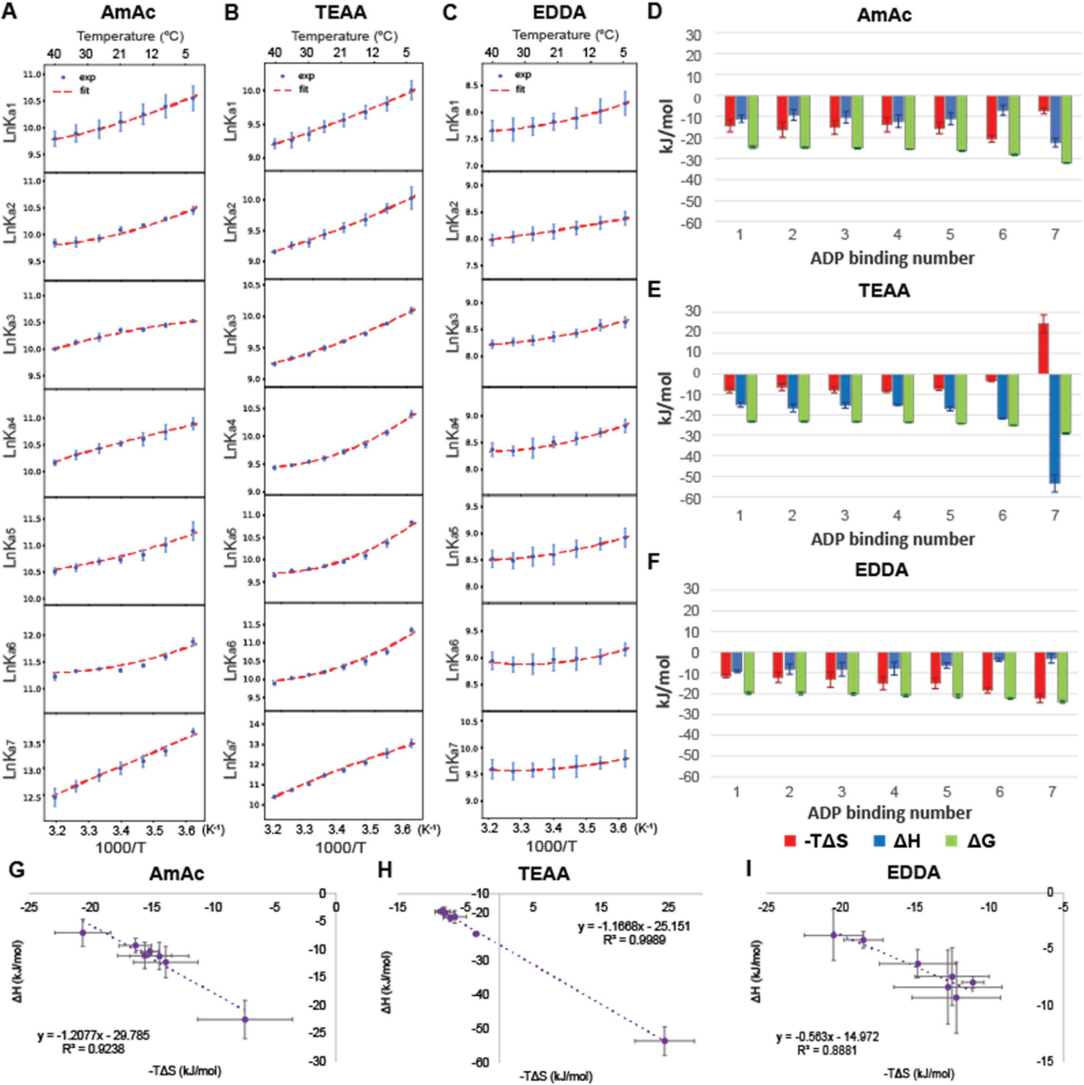
Van’t Hoff fitting plots are shown in (A) 200 mM AmAc, (B) 200 mM TEAA, and (C) 200 mM EDDA. The corresponding entropy, enthalpy, and free-energy values for individual ADP binding steps at 25 °C are shown in bar charts in (D–F), respectively. Plots comparing EECs in (G) 200 mM AmAc, (H) 200 mM TEAA, and (I) 200 mM EDDA. The slope of the fitted line is close to unity; a perfect EEC should have a slope of 1. All values are generated from triplicated data sets and error bars are standard deviations of the three replicates.

The van’t Hoff plots show noticeable nonlinear behavior for individual binding steps, where the slope inflection points are at ca. 22 °C, similar to Δ*Z*_avg_. In AmAc, the first 2 ADP bindings show convex van’t Hoff plots, then the third and fourth ADP bindings show more linear curves, and then the fifth and sixth ADP binding plots become convex again, whereas the plot for the seventh ADP binding becomes linear. In TEAA, van’t Hoff plots for the fifth–seventh ADP bindings are similar to the corresponding binding steps in AmAc; however, the plots for the first 3 ADP bindings are linear instead of convex, whereas the fourth ADP plot is more convex than in AmAc. In EDDA, all binding steps correspond to convex van’t Hoff plots. The curvatures of van’t Hoff plots are representative of heat capacity changes (Δ*C*_p_), which are reported in Table S3.

## Discussion

The ability of proteins to populate different, dynamically interconverting conformational protonation microstates underpins the functional roles that these biomolecules play in living systems. The distribution and interconversion of protonation microstates are determined by the free-energy landscape available to the protein (defined by the environment including temperature, pressure, pH, metals, osmolytes, and other biomolecules). At the same time, these protonation microstates and their dynamics are major contributors to the conformational entropy of a functional protein.^73–76^

For native ESI-MS experiments, *Z*_avg_ directly reports on the population shifts of protonation microstates in each individual species, as charge states are determined by protein conformation.^59^ In the case of ADP binding to SR1, temperature-dependent *Z*_avg_ changes in AmAc solutions at temperatures between 5 and 20 °C followed by marked increases in *Z*_avg_ between 20 and 35 °C are evidence for biphasic restructuring of the complex. This restructuring of the complex may reflect cold-induced compaction or reduced hydration below 20 °C, followed by an extension increasing solvent exposure after 20 °C. Moreover, the temperature range over which the *Z*_avg_ changes occur corresponds to well-known effects of changes in hydration.^45,46,77^ Restructuring induced by changes in hydration are consistent with the two-state model for the water structure that has been attributed to cold denaturation of proteins.^78–81^ Temperature-dependent *Z*_avg_ for SR1 decrease in EDDA and TEAA solutions, and the SR1-ADP_*n*_ complexes exhibit larger *Z*_avg_ shifts than SR1, which suggests that EDDA and TEAA ions interact with surface-exposed hydrophilic sites, altering the surface charges and hydration that stabilize SR1. At higher temperatures, SR1 dissociates into smaller oligomers (Figure S2) and the SR1 protonation microstates that are more prone to thermal dissociation are more populated in TEAA. The CCS for both apo- and ADP-bound SR1 are larger in EDDA than in AmAc and TEAA, further evidence that EDDA promotes the restructuring of both apo- and SR1-ADP complexes.^82–84^ The larger CCS in EDDA is consistent with known effects of shape factors, restructuring from spherical to prolate structures.^53,85^ In fact, *Z*_avg_ for SR1-ADP_6–7_ are lower than SR1-ADP_0–5_ across all measured temperatures in AmAc and TEAA, suggesting that the addition of ADP shifts the distribution of protonation microstates, favoring those of extended conformation. Notably, in AmAc and TEAA, changes in *Z*_avg_ indicate conformational shifts that are not evident in CCS, suggesting that the *Z*_avg_ shifts arise from redistribution among protonation microstates rather than large-scale conformational transitions. As shown in prior computational work, changes in surface electrostatics and solvation can lead to shifts in the population of protonation microstates without necessarily perturbing the global fold of the protein.^59^ Meanwhile, differences in ADP-bound and apo-SR1 CCS are less prominent, suggesting that changes in *Z*_avg_ can be used as a sensitive indicator of the induced shifts in the SASA for ligand-driven protein conformational changes.

SR1-ADP binding (Figure 2) is more favorable at low temperatures in both AmAc and TEAA solutions compared to that in EDDA solutions. Also, in AmAc and TEAA, where the binding of ADP leads to a charge state decrease of SR1, the binding affinities are higher and show greater positive cooperativity in contrast to EDDA. Collectively, changes in *Z*_avg_ and ADP binding demonstrate that alkylammonium ions alter the SR1 conformations and stabilities. In fact, these data are highly consistent with the notion that SR1 complexes exist as a population of protonation microstates in solution. Moreover, the relative populations of protonation microstates (proportional to the oligomer conformational entropy) are dictated by temperature, pressure, pH, and hydration, especially the hydration shell (outer water structure), and play critical roles in protein conformation and dynamics.^21,79^ The distinct behaviors observed across buffers likely arise from differences in how the buffer ions affect the water structure. EDDA, a multivalent chelating agent, likely acts like a kosmotrope, promoting a more structured hydration shell that limits conformational shifts required for ADP-induced extension of the apical domain.^86^ In contrast, the monovalent ammonium ions in AmAc and TEAA are more weakly hydrated and less structured, resulting in a dynamic hydration shell permitting greater conformational sampling. It thus seems reasonable to propose that AmAc, EDDA, and TEAA likely exert distinct effects on the water structure, modulating the free-energy landscapes of the SR1 ring in different ways.

Nonlinear van’t Hoff plots are signatures of heat capacity changes (Δ*C*_p_), which often reflect alterations in the hydration of protein, ligands, and other solutes due to conformation and water property changes.^87–90^ Gruber et al. previously observed a nonlinear van’t Hoff plot for SR1-ATP binding, attributing it to the exposure of SR1 hydrophobic surface area.^90^ Compared to their strongly nonlinear ATP binding data,^90^ the curvatures of the van’t Hoff ADP binding plots reported here are much smaller (Figure 3A–C), suggesting that the conformational differences between the ADP-modulated protonation microstates are less pronounced than those induced by ATP, consistent with prior findings of a less expanded GroEL-ADP_7_ structure.^85^ The van’t Hoff plot curvatures vary across individual binding steps, with the fifth - sixth binding step in AmAc and fourth - sixth in TEAA showing notable nonlinearity and suggesting a greater temperature impact on the corresponding SR1-ADP_*n*_ complexes. The turning point for those curves falls within the temperature range of 20–25 °C, where the charge states (Figure 1A) shift owing to changes in hydration.^78–81^ At the same time, weak interactions between the buffer molecules and the protein surface may also be temperature-dependent, as reported by Tanase et al.^91^ and Bezerra et al.^92^ Δ*C*_p_ reflects differences in the extent of molecular motions and solvent interactions between the free and ligand-bound states, including alterations in conformational flexibility and reorganization of hydration shells. A positive Δ*C*_p_ indicates that the final state is more dynamic or more solvated, typically due to increased exposure of hydrophobic or flexible regions to water. In the case of SR1-ADP binding, the positive Δ*C*_p_ values are consistent with cryo-EM evidence that shows ADP induces a conformationally heterogeneous state in GroEL, where the apical domains only partially extend, resulting in increased average hydrophobic surface exposure to solvent.^93^ Thus, while the exact source of the nonlinearity cannot be definitely assigned to conformation and hydration changes in SR1 and ADP molecules or alkylammonium ions, the observed variation across individual ADP binding steps highlights distinct ADP-induced microstate distributions of the SR1 ring.

Deconvoluted mass spectra in Figure 4A show that increasing the EDDA concentration in the EDDA/AmAc buffer system reduces the abundance of the SR1-ADP_5–7_ signals. Most notably, in solutions of 10 mM EDDA in 190 mM AmAc ADP binding is diminished, while EDDA concentrations above 100 mM limit binding to a maximum of 3 ligands. This observation provides evidence for a strong inhibitory effect of EDDA on ADP binding. In contrast, TEAA exhibits a much weaker inhibitory effect. The experiment carried out at pH 6.3–7 (Figure 4B), despite showing slightly increased ADP binding affinity at pH 7, suggests that buffer composition, rather than pH, is the primary factor affecting ADP binding. A similar temperature effect on SR1-ADP binding in EDDA at pH 6.3 and 7 (Figure S4) further supports the suggestion that inhibition arises from weak interactions between EDDA and SR1. Since ammonium-based buffers are known to weakly interact with protein surfaces,^94,95^ our results suggest that the relative affinities of SR1 for the ammonium-based buffer ions follow the order of ethylenediammonium > triethylammonium > ammonium, implying that as the EDDA concentration increases, the ethylenediammonium ions outcompete the ammonium ions interacting with SR1, leading to a redistribution of SR1 protonation microstates and altered nucleotide binding behavior. Given that EDDA increases the CCS of SR1, this further suggests that EDDA influences ADP binding by populating protonation microstates with elongated conformations. Meanwhile, the triethylammonium ion exhibits a significant inhibitory effect only when it is the dominant species in solution, suggesting weaker competition between the triethylammonium and ammonium ions.

**Figure 4:**
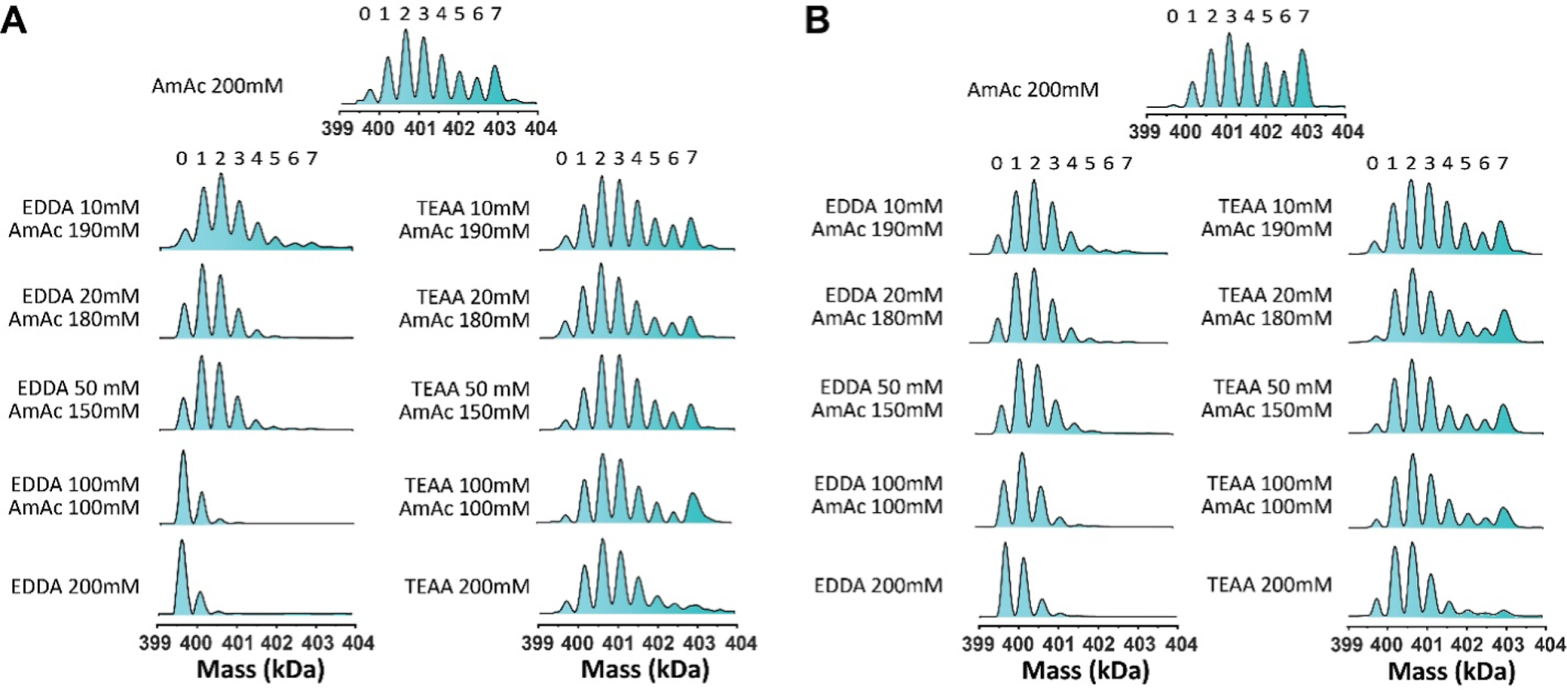
Effects of EDDA and TEAA on SR1-ADP binding in AmAc buffer with increasing concentrations of EDDA or TEAA at (A) the original pH of individual buffers (AmAc at pH 6.8, EDDA at pH 6.3, and TEAA at pH 7) and (B) pH 7 with 1 mM MgAc_2_ and 25 μM ADP at 25 °C. The total buffer concentration is kept at 200 mM. 25 μM ADP was added to SR1, which was prepared in the buffer containing 1 mM MgAc_2_ and buffer molecules with the amounts indicated in each panel.

The EEC profiles for individual ADP binding steps offer additional insights into the thermodynamic behavior and binding patterns of nucleotide-dependent allostery in SR1. Similarity in EEC trends for the first 6 SR1-ADP binding reactions in AmAc and EDDA suggests that they share similar binding mechanisms. Their favorable binding entropies indicate an increasing disturbance of SR1 conformation and hydration. Given that the *Z*_avg_ data suggest minimal ADP-binding-induced restructuring during the binding of the first 5 ADPs, it is reasonable to conclude that binding entropy for the first 6 ADP binding results from an expansion of the underlying ring microstate distribution. As ADP binding shifts this distribution, higher affinity states are more readily populated in AmAc when 6 ADPs are bound, explaining the massive EEC alteration at the seventh binding. In contrast, the binding of the first 6 ADPs in EDDA caused limited changes in SR1 conformation, which results in the EEC of the seventh ADP binding step resembling that of the first 6 steps. This behavior mirrors the moderate decrease in *Z*_avg_ for SR1-ADP_6–7_ at high temperatures (Figure 1E). In TEAA, the first 6 binding steps are primarily enthalpy-driven with much less favorable entropy, suggesting a different binding mechanism. The seventh binding EEC shows a striking difference and is correlated with the conformational change indicated by the *Z*_avg_ decrease for SR1-ADP_6–7_ in Figure 1C.

## Conclusions

In our previous study of wild-type (wt) GroEL, the effect of ESI buffer molecules on nucleotide binding and hydrolysis was highlighted.^33^ Here, we have used vT-nESI mass spectrometry to characterize the properties of different, previously identified SR1 protonation microstates^58^ in the 3 commonly used ESI buffers. As reported by changes in (1) *Z*_avg_, (2) ADP binding affinity, and (3) ADP binding thermodynamics, we found that the distribution of SR1 protonation microstates is altered by different buffer molecules. We also observe different restructurings of the SR1 oligomer (based on *Z*_avg_ analysis) in the 3 buffers. ADP binding thermodynamics (including EEC and nonlinear van’t Hoff plots) suggest that observed alterations in the ADP binding and cooperativity result from shifts in the underlying microstate distributions. While not readily distinguishable by CCS analysis, the conformational differences of these SR1 protonation microstates can be clearly identified by their *Z*_avg_ measurements. Ammonium ion-based mobile-phase buffers are widely utilized for their ion-pairing capabilities in the liquid chromatography analysis of oligonucleotides and peptides, and alkylamine-derived protic ionic liquids coating the stationary phase provide additional weak interactions to improve separations.^95–98^ Our results further demonstrate that the ion-pairing effects of nESI buffer molecules can promote profound changes in the protonation microstate distribution. In particular, the minimized temperature impact on SR1 *Z*_avg_ and inhibited ADP binding in EDDA suggest that the ethylenediammonium ions interact more strongly with SR1. The bimodal “melting curves” for both SR1 and the GroEL tetradecamer in AmAc solutions are consistent with a two-state model for the water structure that interacts with protein surfaces and has been described to explain the cold-denaturation of proteins. Moreover, it is interesting to compare the low affinity, noncooperative ADP binding observed for wt GroEL in AmAc,^33^ whereas SR1 binds ADP with positive cooperativity and substantially higher affinity. These differences suggest that the inter-ring interactions in the wt GroEL restrict the conformational dynamics of individual rings.

Multiple ligand-binding proteins are frequently allosteric, among which GroEL is archetypal.^60,99^ Cooper and Dryden brought new focus on allostery in the 1984 paper “Allostery Without Conformational Change,”^100^ but Nussinov and Tsai questioned this claim and emphasized that the redistribution of protein states coevolves with ligand binding.^101^ This latter view is now widely referred to as dynamic allostery, which is characterized by small changes accompanying the binding of the allosteric effector that are too small to be detected,^102^ viz., allostery without measurable conformational change. VT-nESI-IMS-MS reports changes in *Z*_avg_, CCS, and thermodynamics for binding of “one ligand at a time”.^33^ These changes are signatures for restructuring of the complex, which are interpreted here as a redistribution of protonation microstates. VT-nESI-IMS-MS enables the examination of SR1-ADP binding from the three standpoints suggested by Tsai and Nussinov (thermodynamics, free-energy landscape of population shift, and structure)^103^ and serves as a useful methodology for furthering our understanding of dynamic allostery.

## Supplementary Material



## Notes

Mass assignment of SR1-ADP_*n*_ complexes, deconvoluted mass spectra of SR1-ADP binding at high ADP concentration in EDDA buffer, binding constants of SR1-ADP_*n*_ complexes adjusted for nonspecific binding in three buffers, raw mass spectra of SR1 thermal denaturation in three buffers, ion mobility profiles, comparison of temperature effect on SR1-ADP binding in EDDA at pH 6.3 and 7, thermodynamic profile for SR1-ADP_n_ complexes in EDDA adjusted for nonspecific binding, and heat capacity changes (PDF)
