# Supplementary material for: Allostery without Conformational Change: A Native Mass Spectrometry Perspective
Source: J Phys Chem B. 2025 Aug 19;129(34):8668–79. doi: 10.1021/acs.jpcb.5c03261 (PMC12400411; doi:10.1021/acs.jpcb.5c03261)
Supplement: Supplementary file 1 [file jp5c03261_si_001.pdf]

Supporting Information

Allostery Without Conformational Change: A Native Mass Spectrometry Perspective

He Mirabel Sun,[a] Kacie A. Evans,[a] Morgan Powers,[b] Zhenyu Xi,[a] Carter Lantz,[a] Arthur Laganowsky,[a] Hays Rye[b]*, David H. Russell[a]*

[a]Department of Chemistry, Texas A&M University, College Station, Texas 77843

[b]Department of Biochemistry and Biophysics, Texas A&M University, College Station, Texas 77843

*Email: hays.rye@ag.tamu.edu.

*Email: russell@chem.tamu.edu.

Table of Contents

Page

Table S1. Deconvoluted mass of SR1-ADP$_n$ in each buffer of study S2

Table S2. Full width half maximum of the deconvoluted MS peaks of SR1-ADP$_n$ in each buffer of study S2

Figure S1. Deconvoluted mass spectrum of SR1-ADP$_n$ in EDDA with 300 μM ADP S3

Table S3. Binding constants of SR1-ADP$_n$ in each buffer adjusted for nonspecific binding S3

Figure S2. Comparison of mass spectrum showing buffer effect on SR1 thermal dissociation at 45 °C S4

Figure S3. Ion mobility profiles of apo and ADP-bound SR1 in each buffer of study S5

Figure S4. Deconvoluted mass spectra comparing the ADP binding thermodynamics in EDDA at pH 6.3 and pH 7 S5

Figure S5. Thermodynamic profile for SR1-ADP$_n$ in EDDA accounting for nonspecific binding S6

Table S3. Values of heat capacity change (ΔCp, kJ·mol^{-1}·K^{-1}) for individual binding steps in each buffer of study S6

Table S1. Deconvoluted mass (kDa) of SR1-ADP$_n$ in different buffers. Δmass represents an increase in mass as the addition of ADP+Mg^{2+} is bound for each species. The values are the averages of triplicated data sets.

	**AmAc**		**EDDA**		**TEAA**	
	Mass (kDa)	ΔMass (Da)	Mass (kDa)	ΔMass (Da)	Mass (kDa)	ΔMass (Da)
SR1 (apo)	399.73		399.57		399.78	
SR1-ADP$_1$	400.19	461	400.03	460	400.24	463
SR1-ADP$_2$	400.64	459	400.49	459	400.70	459
SR1-ADP$_3$	401.10	463	400.93	462	401.17	471
SR1-ADP$_4$	401.57	472	401.38	456	401.63	461
SR1-ADP$_5$	402.03	467	401.82	447	402.09	463
SR1-ADP$_6$	402.48	458	402.27	453	402.55	462
SR1-ADP$_7$	402.92	448	402.73	462	403.03	476

Table S2. Full width half maximum for the peaks of the deconvoluted mass spectra of SR1-ADP$_n$ in different buffers (kDa).

	**AmAc**	**EDDA**	**TEAA**
SR1 (apo)	0.21	0.17	0.22
SR1-ADP$_1$	0.23	0.19	0.25
SR1-ADP$_2$	0.23	0.18	0.25
SR1-ADP$_3$	0.23	0.22	0.24
SR1-ADP$_4$	0.22	0.22	0.26
SR1-ADP$_5$	0.22	0.23	0.25
SR1-ADP$_6$	0.23	0.23	0.29
SR1-ADP$_7$	0.30	0.25	0.35

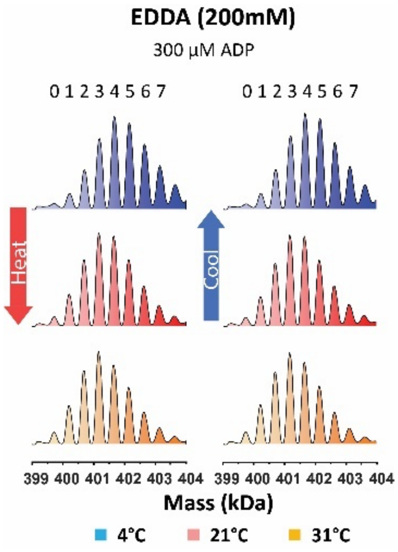

Figure S1. Deconvoluted mass spectra of ADP binding products of 1 μM SR1 in 200 mM AmAc, 200 mM EDDA, and 200 mM TEAA buffer containing 1 mM $MgAc_2$ and 300 μM ADP at cold (4°C), medium (21°C), and high (31°C) temperatures.

Table S3. Intrinsic binding constants (Ka) for individual SR1-ADP binding steps at 4 °C, 21 °C, and 31 °C in AmAc, TEAA, and EDDA adjusted to account for nonspecific binding. The "nonspecific" label under ADP Binding Number refers to the nonspecific association binding constant. It was determined that the 5th-7th ADP binding events in EDDA were nonspecific and therefore are not reported here.

ADP Binding Number	AmAc			TEAA			EDDA		
	4 °C	21 °C	31 °C	4 °C	21 °C	31 °C	4 °C	21 °C	31 °C
1	0.0340	0.0222	0.0185	0.0211	0.0135	0.0109	0.0032	0.0024	0.0022
2	0.0330	0.0241	0.0199	0.0216	0.0133	0.0101	0.0037	0.0028	0.0025
3	0.0366	0.0288	0.0228	0.0224	0.0136	0.0108	0.0041	0.0028	0.0026
4	0.0506	0.0333	0.0278	0.0306	0.0151	0.0120	0.0050	0.0034	0.0029
5	0.0753	0.0436	0.0379	0.0472	0.0187	0.0150	NA	NA	NA
6	0.1385	0.0856	0.0841	0.0798	0.0305	0.0234	NA	NA	NA
7	0.8895	0.4522	0.3332	0.8812	0.2123	0.0970	NA	NA	NA
Nonspecific	0.0073	0.0068	0.0067	0.0091	0.0068	0.0077	0.0049	0.0042	0.0039

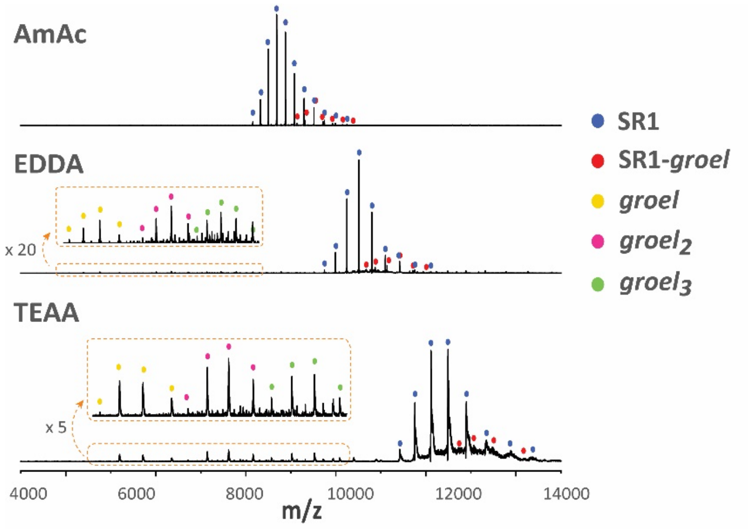

Figure S2. Comparison of signals in mass spectra of SR1 and thermal dissociation products at 45°C in AmAc, EDDA, and TEAA buffer, respectively. The term *groel* refers to the single subunit of SR1.

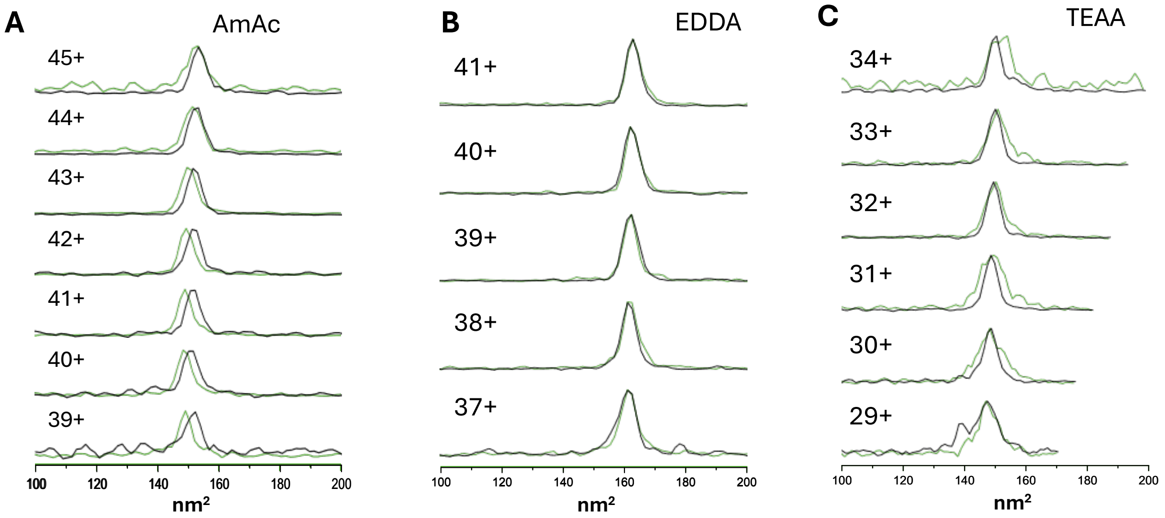

Figure S3. The collisional cross section of SR1 in the absence of ADP (black trace) and with 100 μM ADP (green trace) at individual charge states in **(A)** 200 mM AmAc, **(B)** 200 mM EDDA buffer, and **(C)** 200 mM TEAA buffer at 25°C. The samples all contain 1mM $MgAc_2$ and 1μM SR1.

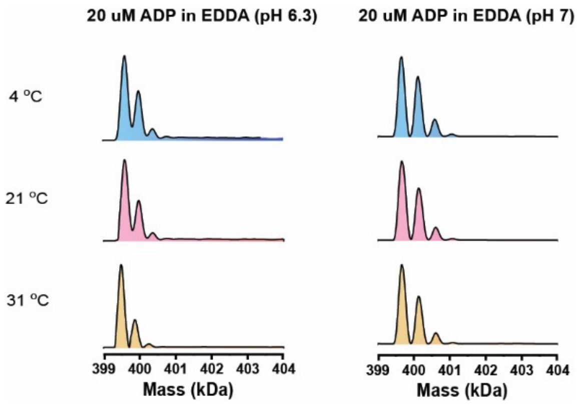

Figure S4. Comparison of deconvoluted mass spectra of ADP binding products of 1 μM SR1 in 200 mM AmAc, 200 mM EDDA, and 200 mM TEAA buffer containing 1 mM $MgAc_2$ and 20 μM ADP at cold (4°C), medium (21°C), and high (31°C) temperatures in pH 6.3 vs pH 7.

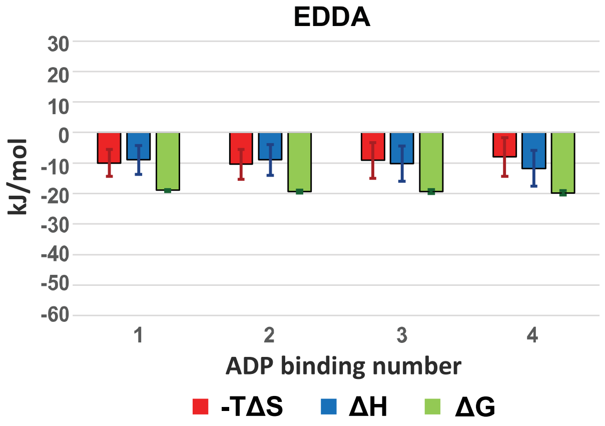

Figure S5. Bar chart showing the entropy, enthalpy, and free energy values for individual ADP binding steps at 25 °C adjusted to account for nonspecific binding. All values are generated from triplicated data sets and error bars are the standard deviation of the three replicates.

Table S3. Values of heat capacity change (ΔC_p, unit in $kJ{\cdot}mol^{-1}{\cdot}K^{-1}$) in AmAc, TEAA, and EDDA buffer for individual ADP binding steps, respectively. All values are obtained through van't Hoff analysis shown in **Figure 3**, generated from triplicated data sets with corresponding R^2 reported.

	AmAc	R^2	TEAA	R^2	EDDA	R^2
1	0.27±0.23	1.00	0.27±0.25	1.00	0.39±0.34	1.00
2	0.25±0.40	0.98	0.39±0.42	1.00	0.08±0.16	1.00
3	-0.25±0.38	0.97	0.43±0.22	0.99	0.27±0.16	1.00
4	-0.03±0.19	0.99	1.04±0.25	0.99	0.20±0.09	1.00
5	0.54±0.18	0.98	1.38±0.03	0.99	0.37±0.17	0.99
6	0.45±0.10	0.95	1.29±0.47	0.84	0.23±0.16	0.98
7	0.06±0.04	1.00	-0.11±0.61	0.99	0.34±0.21	1.00
